# Hepatitis C Virus Infection Among Women Giving Birth — Tennessee and United States, 2009–2014

**DOI:** 10.15585/mmwr.mm6618a3

**Published:** 2017-05-12

**Authors:** Stephen W. Patrick, Audrey M. Bauer, Michael D. Warren, Timothy F. Jones, Carolyn Wester

**Affiliations:** ^1^Departments of Pediatrics and Health Policy, Division of Neonatology, Vanderbilt University School of Medicine, Nashville, Tennessee; ^2^Tennessee Department of Health.

Hepatitis C virus (HCV) affects an estimated 3.5 million persons in the United States (*1*), making it the most common bloodborne infection in the country. Recent surveillance data showed increased rates of HCV infection among adolescents and adults who are predominantly white, live in nonurban areas, and have a history of injection drug use.* U.S. birth certificate data were used to analyze trends and geographic variations in rates of HCV infection among women giving birth during 2009–2014. Birth certificates from Tennessee were used to examine individual characteristics and outcomes associated with HCV infection, using a multivariable model to calculate adjusted odds of HCV-related diagnosis in pregnancy among women with live births. During 2009–2014, HCV infection present at the time of delivery among pregnant women from states reporting HCV on the birth certificate increased 89%, from 1.8 to 3.4 per 1,000 live births. The highest infection rate in 2014 (22.6 per 1,000 live births) was in West Virginia; the rate in Tennessee was 10.1. In adjusted analyses of Tennessee births, the odds of HCV infection were approximately threefold higher among women residing in rural counties than among those in large urban counties, 4.5-fold higher among women who smoked cigarettes during pregnancy, and nearly 17-fold higher among women with concurrent hepatitis B virus (HBV) infection. HCV infection among pregnant women is an increasing and potentially modifiable threat to maternal and child health. Clinicians and public health officials should consider individual and population-level opportunities for prevention and risk mitigation.

Data from 2009–2014 were obtained from the National Vital Statistics System and Tennessee Department of Health vital records. The outcome of interest was HCV infection in pregnant women at the time of delivery (maternal HCV infection) as indicated on the infant’s birth certificate. The maternal HCV infection rate per 1,000 deliveries in Tennessee was compared with that from hospital billing data in the Tennessee Hospital Discharge Data System, an all-payer administrative database that includes data for all inpatient admissions in the state. National data were compared with nationally weighted estimates obtained from the National Inpatient Sample, the largest all payer database in the United States.^†^

The first phase of the analysis examined rates of maternal HCV infection reported on infant birth certificates to approximate HCV infection among pregnant women in the United States. Because HCV infection is a revised 2003 birth certificate item, states gradually reported this item over time as they adopted the revised certificate; therefore, rates were calculated based on records from all states^§^ with available data at any time during 2009 and 2014.^¶^ The second phase of the analysis used data from Tennessee vital records to assess sociodemographic characteristics, gravidity, health behaviors, and other infections during pregnancy associated with HCV infection in pregnancy. Overall, <1% of data for variables included in the study were missing, with the exception of timing of prenatal care, which was missing for 6.2% of records. To account for missing data, multiple imputation using chained equations with 20 imputations was used. A multivariable logistic regression model was fit to the data to determine increased odds of HCV infection in pregnancy, simultaneously adjusting for maternal age, education, marital status, race/ethnicity, county of residence, number of previous pregnancies, late or no prenatal care, smoking during pregnancy, and other infections present at delivery, including chlamydia, gonorrhea, syphilis, herpes simplex virus, and HBV. The statistical significance level was set to p<0.05 for all tests. The study was approved by the Tennessee Department of Health’s institutional review board.

During 2009–2014, the prevalence of maternal HCV infection among reporting states increased 89%, from 1.8 to 3.4 per 1,000 live births (p<0.001). There was substantial state-to-state variation in maternal HCV rates: in 2014, the highest rate (22.6 per 1,000 live births) was in West Virginia, and the lowest (0.7) was in Hawaii (Figure 1). In Tennessee, the prevalence of maternal HCV infection increased 163%, from 3.8 per 1,000 live births in 2009 to 10.0 in 2014 (p<0.001). Within Tennessee, there was substantial variation among 95 counties, with the highest rates in the 52 Appalachian counties in the eastern part of the state. For example, in 2014, Campbell County had the highest rate in Tennessee (78 per 1,000 births); 19 other counties had rates of ≤1 per 1,000 births, including 18 counties that reported no cases (Figure 2). Analysis of maternal HCV infection rates based on hospital discharge data resulted in similar findings.

**FIGURE 1:**
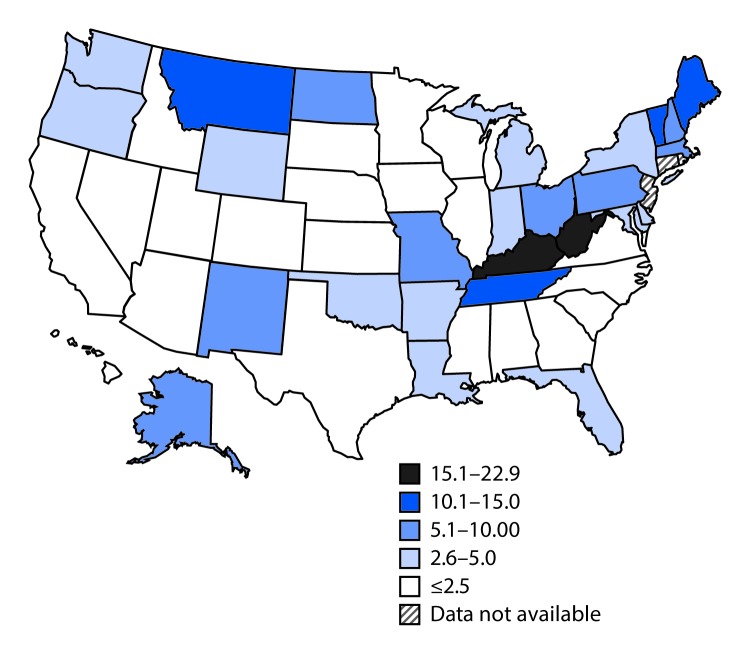
Rate of hepatitis C infection among pregnant women per 1,000 live births, by state — United States, 2014

**FIGURE 2:**
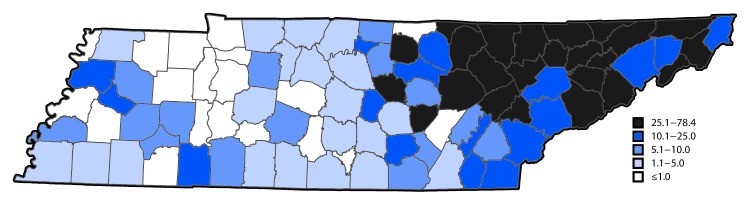
Rate of hepatitis C infection among pregnant women per 1,000 live births, by county — Tennessee, 2014

In adjusted analyses of Tennessee births from 2009 to 2014, compared with women without HCV infection, women with diagnosed HCV at the time of live birth had higher odds of having a high school education or less, being unmarried, having late or no prenatal care, and smoking cigarettes. Compared with pregnant non-Hispanic white women, non-Hispanic black women had nearly 80% lower odds, and Hispanic women nearly 70% lower odds of having a diagnosis of HCV. Residing in a rural county was also associated with higher odds of maternal HCV infection. When compared with large central metro areas (counties with >1,000,000 population), the odds of HCV infection among pregnant women from rural areas (counties with <50,000 population) were threefold higher. Concurrent infections also were associated with higher odds of having an HCV diagnosis, with HBV infection resulting in nearly 17-fold increased odds of HCV (Table).

**TABLE:** Adjusted maternal characteristics associated with hepatitis C infection at the time of birth — Tennessee, 2009–2014

Characteristic	Adjusted odds ratio (95% CI)
**Age/Education/Marital status**	
Older age	1.05 (1.04–1.06)
High school graduate or less	1.90 (1.74–2.08)
Unmarried	2.12 (1.95–2.31)
**Race/Ethnicity**	
White, non-Hispanic	referent
Black, non-Hispanic	0.23 (0.19–0.27)
Hispanic	0.33 (0.26–0.41)
Other	0.61 (0.43–0.87)
Classification of residence county*	
Large central metro	referent
Large fringe metro	1.21 (0.99–1.48)
Medium metro	4.38 (3.72–5.15)
Small metro	4.65 (3.88–5.56)
Micropolitan	3.05 (2.56–3.64)
Noncore	3.07 (2.55–3.69)
**Pregnancy**	
One or more previous pregnancies	1.58 (1.44–1.74)
Late or no prenantal care	1.74 (1.61–1.88)
Smoked during pregnancy	4.49 (4.13–4.89)
**Infections during pregnancy**	
Chlamydia	1.35 (1.13–1.61)
Gonorrhea	1.67 (1.13–2.48)
Syphilis	1.57 (0.72–3.43)
Herpes simplex virus	1.96 (1.74–2.21)
Hepatitis B	16.60 (12.70–21.68)

**Abbreviation:** CI = confidence interval.

* Maternal residence county was classified using the 2013 National Center for Health Statistics Urban–Rural Classification Scheme. *Large central metro* = Counties in metropolitan statistical areas of ≥1 million population that 1) contain the entire population of the largest principal city of the metropolitan statistical area (MSA), or 2) have their entire population contained in the largest principal city of the MSA, or 3) contain at least 250,000 inhabitants of any principal city of the MSA. *Large fringe metro* = Counties in MSAs of ≥1 million population that did not qualify as large central metro counties. *Medium metro* = Counties in MSAs with populations of 250,000–999,999. *Small metro* = Counties in MSAs with populations of <250,000. *Micropolitan* = Counties in micropolitan statistical areas with populations of 10,000–49,999. *Noncore* = Nonmetropolitan counties that did not qualify as micropolitan.

^†^ A multivariable logistic regression model was fit to the data to determine increased odds of HCV infection in pregnancy, simultaneously adjusting for maternal age, education, marital status, race/ethnicity, county of residence, number of previous pregnancies, late or no prenatal care, smoking during pregnancy, and other infections present at delivery, including chlamydia, gonorrhea, syphilis, herpes simplex virus and hepatitis B virus infection.

## Discussion

From 2009 to 2014, the prevalence of HCV infection among U.S. women giving birth in reporting states nearly doubled. This increase in maternal HCV infection mirrors increases in HCV infection incidence among adults, particularly nonpregnant young adults in the United States. A recent study identified a similar increase in HCV prevalence among women with recent live births (*2*); this study builds upon those findings, identifying several patient-level characteristics associated with maternal HCV infection, including white race, rural county residence, cigarette smoking during pregnancy, having a high school education or less, and having a concurrent HBV infection. In the United States, CDC and the American College of Obstetricians and Gynecologists recommend selective screening of pregnant women at high risk for HCV infection (i.e., history of injection drug use or long-term hemodialysis) (*3*). These data might inform expansion of the definition of women at risk, thereby improving clinical detection, particularly in areas of a state reporting increasing or high rates of incident HCV infection.

The recent increase in maternal HCV infection appears to have disproportionately affected rural and white populations; states and Tennessee counties with the highest prevalence of HCV infection among pregnant women in 2014 were in predominately Appalachian regions.** A recent analysis of state surveillance data examining acute HCV infections in the general population found a near doubling of cases in the United States during 2006–2012, and also found that states in or near Appalachian regions had the highest numbers of cases (*4*), suggesting that primary prevention and testing and treatment strategies for HCV infection could be targeted to these populations and areas at high risk.

This increase in HCV infection is particularly concerning in light of recent research highlighting poor follow-up of HCV-exposed infants (*5*). The rate of vertical transmission from infected mothers to infants is estimated at 6% (11% if the mother is coinfected with human immunodeficiency virus [HIV]) (*6*); therefore, it is important that exposed infants be followed for evidence of seroconversion. Because passively acquired maternal antibodies can persist for up to 18 months, anti-HCV antibody tests cannot be completed until that time; however, testing for HCV RNA can be performed earlier (*7*). A recent study in Philadelphia found that only 16% of HCV-exposed infants were appropriately followed (*5*), suggesting that infected infants might go undetected.

The increase in maternal HCV infection coincides with the rising heroin and prescription opioid epidemics occurring in the United States that have also disproportionately affected rural and white populations (*8*,*9*). There has also been a recent surge in opioid use among pregnant women (*8*). Whereas HCV infections have historically been associated with heroin use, a recent outbreak of HIV and HCV in rural Indiana demonstrates that these infections can also be transmitted through use of injectable forms of prescription opioids (*10*).

The findings in this report are subject to at least three limitations. First, vital records data rely on accurate coding of birth certificates; some variables such as HCV might be undercoded, and misclassification bias might occur. However, evaluation of hospital administrative data reporting of HCV infections suggests that this effect is small. Second, the proportion of live births from which data were collected on HCV status increased during the study period, as more states adopted the revised certificate each year. Because the original reporting states in 2009 were not held constant over time for this analysis, it is possible the trend could be subject to ascertainment bias; however, two additional confirmatory analyses were performed: 1) comparison with the National Inpatient Sample demonstrated similar rates of HCV infection during 2009–2013 (1.8 per 1,000 to 3.1 per 1,000), and 2) the same trend analysis was performed holding the original 28 reporting states in 2009 constant. Results were the same as when using all 47 states that incorporated reporting over time. Because women are not universally screened for HCV in pregnancy, these estimates and analyses do not represent the actual prevalence of HCV in pregnant women. However, the findings of increased disease prevalence among white and rural populations are similar to those of recent studies in nonpregnant populations (*4*). Instances of multiple births might have resulted in a slight overestimation of rates of maternal HCV infection. Finally, it is important to consider that HCV infections in a given state might represent not only the prevalence of a condition but also the public health efforts implemented to detect and treat the infection.

The prevalence of maternal HCV infection appears to have increased sharply in the United States, presenting concerns for maternal and child health. Ensuring that women of childbearing age have access to HCV testing and treatment and consideration of universal screening among women of reproductive age residing in areas with high HCV prevalence might mitigate risk and prevent transmission.

**Summary**

**What is already known about this topic?**

Hepatitis C virus (HCV) infection affects approximately 3.5 million persons in the United States, making it the most common bloodborne infection in the nation. Recent surveillance data demonstrate increased rates of HCV infection among adolescents and adults who are predominantly white, live in nonurban areas, and have a history of injection drug use.

**What is added by this report?**

During 2009–2014, maternal HCV infections nearly doubled among reporting states in the United States, with substantial state-to-state variation in prevalence. In adjusted analyses of Tennessee births, residence in a rural county was associated with a more than threefold increase in the odds of maternal HCV infection. Smoking during pregnancy and concurrent hepatitis B virus infection imparted fourfold and nearly 17-fold increased odds of maternal HCV infection, respectively.

**What are the implications for public health practice?**

Screening for HCV infection in women of childbearing age and provision of treatment services might reduce perinatal transmission of HCV, and monitoring of HCV-exposed infants can aid in early identification of HCV infection and related liver disease.

## Footnotes

* https://www.cdc.gov/hepatitis/statistics/2014surveillance/index.htm.

† Maternal records were included in the analysis if they had one or more *International Classification of Diseases, Ninth Revision, Clinical Modification* (ICD-9-CM) diagnostic and procedure codes or Diagnosis Related Groups indicating delivery. HCV-positive women were identified using the following ICD-9-CM codes: 070.41, 070.44, 070.51, 070.54, 070.7, 070.70, and 070.71.

§ Data were available for all states except Connecticut, New Jersey, and Rhode Island. Data also were not available for the District of Columbia.

¶ Births records where HCV status was either unknown or not collected were excluded from national estimates. Percentage of total births excluded for each year: 31.9% (2009), 22.6% (2010), 14.2% (2011), 11.7% (2012), 9.6% (2013), and 3.6% (2014).

** https://www.arc.gov/counties.
